# Periods of high dengue transmission defined by rainfall do not impact efficacy of dengue vaccine in regions of endemic disease

**DOI:** 10.1371/journal.pone.0207878

**Published:** 2018-12-13

**Authors:** Chloé Pasin, M. Elizabeth Halloran, Peter B. Gilbert, Edith Langevin, R. Leon Ochiai, Punnee Pitisuttithum, Maria Rosario Capeding, Gabriel Carrasquilla, Carina Frago, Margarita Cortés, Laurent Chambonneau, Zoe Moodie

**Affiliations:** 1 Université de Bordeaux, INSERM U1219 Bordeaux Population Health center, INRIA SISTM, Bordeaux, France; 2 Vaccine Research Institute, Creteil, France; 3 ENS Cachan, Université Paris-Saclay, Cachan, France; 4 Fred Hutchinson Cancer Research Center, Seattle, Washington, United States of America; 5 University of Washington, Seattle, Washington, United States of America; 6 Sanofi Pasteur, Marcy L’Etoile, France; 7 Sanofi Pasteur, Singapore, Singapore; 8 Vaccine Trial Centre and Department of Clinical Tropical Medicine, Faculty of Tropical Medicine, Mahidol University, Nakorn Pratum, Thailand; 9 Research Institute for Tropical Medicine, Alabang, Muntinlupa City, Philippines; 10 Fundación Santa Fe de Bogotá, Bogotá, Colombia; 11 Sanofi Pasteur, Bogotá, Colombia; Faculty of Science, Ain Shams University (ASU), EGYPT

## Abstract

**Objective:**

To evaluate the association of rainy season with overall dengue disease incidence and with the efficacy of the Sanofi Pasteur recombinant, live, attenuated, tetravalent vaccine (CYD-TDV) in two randomized, controlled multicenter phase III clinical trials in Asia and Latin America.

**Methods:**

Rainy seasons were defined for each study site using climatological information from the World Meteorological Organization. The dengue attack rate in the placebo group for each study month was calculated as the number of symptomatic, virologically-confirmed dengue events in a given month divided by the number of participants at risk in the same month. Time-dependent Cox proportional hazard models were used to test whether rainy season was associated with dengue disease and whether it modified vaccine efficacy in each of the two trials and in both of the trials combined.

**Findings:**

Rainy season, country, and age were all significantly associated with dengue disease in both studies. Vaccine efficacy did not change during the rainy season in any of the analyses.

**Conclusions:**

Although dengue transmission and exposure are expected to increase during the rainy season, our results indicate that CYD-TDV vaccine efficacy remains constant throughout the year in endemic regions.

## Introduction

Dengue is a mosquito-transmitted viral disease found in tropical and subtropical areas, as well as some temperate regions of the United States, Europe, Africa, and the Middle East [1]. Almost 4 billion people are estimated to be at risk for dengue infection in more than 100 countries around the world [2]. In recent decades, the number of reported dengue cases has dramatically increased, making dengue a disease of global public health importance. Dengue infection is usually asymptomatic but also has a wide spectrum of clinical manifestations. Of the estimated 390 million children and adults infected each year, 96 million exhibit clinical disease [3]. Clinical dengue disease includes influenza-like symptoms, skin rash, and more severe manifestations such as severe plasma leakage, severe bleeding, and severe organ impairment. Four antigenically different serotypes of dengue virus exist; secondary infection with a heterologous serotype has been shown to be a risk factor for severe dengue [4–6].

Dengue prevention consists primarily of measures that aim to control the *Aedes aegypti* or *Aedes albopictus* vector, such as habitat elimination to reduce the risk of mosquito bites [7]. However, these measures have had limited ability to protect against disease as outbreaks still occur frequently. Moreover, there is currently no specific antiviral treatment against dengue, although several dengue vaccines are in development [8]. The recombinant live attenuated tetravalent vaccine (CYD-TDV) developed by Sanofi Pasteur has been tested in two phase III efficacy trials: one placebo-controlled phase III trial of the CYD-TDV dengue vaccine was conducted in 2–14 year olds in South East Asia with the active surveillance phase lasting from June 2011 to December 2013 (CYD14) [9] and another phase III trial conducted in 9–16 year olds in Latin America with the active surveillance phase lasting from June 2011 to April 2014 (CYD15) [10] (the phase III trials are discussed further in [11]). In both trials, after randomization of children to receive vaccine or placebo at 0,6 and 12 months, active surveillance for symptomatic virologically-confirmed dengue (VCD) followed over 25 months after the first injection. The overall intent-to-treat vaccine efficacy (VE) was 54.8% (95%CI 46.8, 61.7) and VE against severe dengue 70% (95%CI 35.7, 86.6) in Asia versus 64.7% (95%CI 58.7, 69.8) and 95.5% (95%CI 68.8, 99.9) respectively in Latin America during this active phase. These findings supported the licensing of CYD-TDV in several dengue endemic countries where the seroprevalence rate is more than 70% [11].

Variation of VE was observed depending on the seroprevalence in the countries: for example, in CYD15, seroprevalence was higher in Brazil and Colombia than in Mexico and Puerto Rico (55.5%, 92.5%, 50.9% and 48.9% respectively), and so was efficacy (77.5%, 67.5%, 31.3% and 57.6% respectively) [10,12]. In exploratory analyses of CYD14 and CYD15, estimated VE was higher for older children and for children who were seropositive to at least one dengue serotype at baseline compared to children who were seronegative [9, 10]. Moreover, during the year 3 of the long-term safety follow-up in CYD14, an excess in the number of dengue hospitalization and/or severe cases was found in the vaccinated younger age group (2 to 5 years old). Sanofi Pasteur performed additional analyses of long term follow up safety data to better understand whether these findings were due to age effect or previous exposure to dengue [13] and to assess the potential risk and/or benefit of vaccination of baseline-seronegative individuals. These analyses indicated that while vaccination in endemic areas confers predicted benefit on the population level, the vaccine performs differently in seropositive vs seronegative individuals, with baseline-seronegative individuals receiving the vaccine showing increased risk of hospitalized and severe dengue compared to placebo recipients, starting about 30 months post-first dose [14]. It is not clear if the possible age effect may be associated with pre-existing dengue exposure or age-specific differences (e.g. less-developed vascular physiology or immature immune responses in the younger age group). The World Health Organization thus concluded that a “‘prevaccination screening strategy’ would be the preferred option, in which only dengue-seropositive-persons are vaccinated [14].” However, no point-of care diagnostic test is currently available that would allow rapid, sensitive, and specific prevaccination screening to determine baseline serostatus; the development of such a test has been outlined as a high research priority [12].

A question of interest is whether VE varies according to dengue transmission intensity. There is a prevailing view in general vaccinology that, in the setting of a “leaky vaccine”, VE may be reduced during periods of high exposure [15]. It has been underlined that in the context of infectious diseases, the temporal pattern of the varying risks of infection should be accounted for in the evaluation of vaccine efficacy, e.g. for the DTaP pertussis vaccine [16], for which vaccine efficacy was suggested to be lower during periods of higher exposure [17]. In an additional study on the RV144 HIV vaccine, a significant effect modification was seen in behavioral risk status over time and VE where VE was 68% for participants who maintained low or medium risk but only 5% for the high risk group [18]. In an earlier trial among Thai injecting drug users, Vax 003, of the same AIDSVAX B/E component as RV144, it is hypothesized that the lack of VE may have been due to the stringency of intravenous challenge when compared with the intravaginal and intrarectal routes of RV144 [19]. In the related disease setting of malaria, concerns have also been raised about whether the transmission intensity of the disease might impact vaccine efficacy [20–22], in particular due to the seasonal variation of the natural force of infection [23]. To address this question, a study in Malawi evaluated effect modification of RTS,S/AS01 malaria vaccine efficacy by seasonal precipitation and found there was none [24]. Similar to malaria transmission, dengue transmission is influenced by many factors [25–28]; climatic factors such as temperature and rainy season can play a particularly important role in shaping dengue epidemics and should be carefully considered when studying these epidemics [29]. Some studies have shown how these factors could be used to predict dengue outbreaks [30,31]. Specifically, several pieces of evidence indicate that dengue transmission is increased at high temperatures. First, *A*. *aegypti* or *A*. *albopictus* reproduction, bite rate, and survival all increase with temperature until a cut-off of approximately 32°C is reached [32–35]. High temperature also shortens the extrinsic incubation period, such that mosquitoes become infectious faster [36,37]. Rainy season has also been found to be associated to dengue transmission [38,39], although the findings can vary between studies and geographic locations [40]. Nevertheless, rain is believed to increase the number of available breeding sites (e.g., water-filled containers) and hence the number of mosquitoes, suggesting increased transmission during the rainy season. We considered rainy season data to provide a better basis than temperature data for defining periods of high dengue intensity due to its direct effect on mosquito habitats. Thus, we used rainy season as a proxy for the force of dengue infection to evaluate the association of rainy season with overall dengue disease and on the efficacy of the CYD-TDV vaccine.

## Methods

### Data

We analyzed data from the two primary phase III clinical trials on which CYD-TDV licensure was based: CYD14, conducted in 10, 275 children in five countries in South East Asia, and CYD15, conducted in 20,869 children in five countries in Latin America. Children in each trial were randomly assigned in a 2:1 ratio to receive three injections of either CYD-TDV or placebo, respectively, at months 0, 6, and 12. Children were followed for symptomatic virologically-confirmed dengue until month 25 after the first injection. During this period, all children were under active surveillance. Surveillance consisted of weekly reminders to the family to go to the health care center if acute febrile illness was observed. In cases of acute febrile illness, two blood samples were collected for virological confirmation of the presence of dengue. One sample was collected within 5 days after the onset of fever and the second one was obtained 7 to 14 days later. The first sample was tested for dengue nonstructural protein 1 (NS1) antigen using an ELISA assay (Platelia Bio-Rad Laboratories, Marnes-La-Coquette, France [41]) and with a quantitative reverse transcription PCR assay and a serotype-specific PCR assay (Simplexa Dengue Real-Time PCR Assay, Focus Diagnostics, CA, USA). If any of these tests were positive, the event was considered to be virologically-confirmed dengue. Both the first and the second samples were tested for dengue IgM and IgG. The first day of the acute febrile illness was used as the date of virologically-confirmed dengue.

### Ethics statement

The trial protocols were approved by all relevant ethics review boards, and parents or guardians provided written informed consent and older children provided written informed assent before participation, in accordance with local regulations. All patient data were anonymized.

The ethics review boards for CYD14 were the following:

- The Committee of Medical research Ethics, Faculty of Medicine, University of Indonesia, Jakarta Indonesia
- The Research and Development Unit Medical Faculty University of Udayana, Sanglah General Hospital, Denpasar, Indonesia
- Health Research Ethics Committee, Faculty of medicine University of Padjadjadrain, Dr Hasan Sadikin hospital, Bandung, Indonesia
- Medical Research and Ethics Committee, Ministry of Health, Malaysia, Kuala Lumpur, Malaysia
- Research institute for Tropical Medicine IRB, Alabang, Muntinlupa City, Philippines
- Vicente Sotto memorial Medical Center EC, Cebu City, Philippines
- Chong Hua Hospital Institutional Review Board, Cebu City, Philippines
- Walter Reed Army Institute of Research international Review Board (WRAIR IRB), Md, USA
- The Ethical Review Committee for Research in human Subjects, Ministry of Public Health, Thailand
- Ethics Committee of the Faculty of Tropical Medicine, Mahidol University, Bangkok, Thailand
- Pasteur Institute EC, Ho Chi Minh City, Vietnam

The ethics review boards for CYD15 were the following:

- Comitê de Ética em Pesquisa do Centro Ciências da Saúde (CCS) da Universidade Federal do Espírito Santo (UFES) (CEP/CCS/UFES)
- Comitê de Ética em Pesquisa em Seres Humanos do Hospital Universitário Onofre Lopes / RN
- Comitê de Ética em Pesquisa em Seres Humanos do Hospital das Clínicas da Universidade Federal de Goiás
- Comitê de Ética em Pesquisa em Seres Humanos da Universidade Federal de Mato Grosso do Sul—UFMS
- Comitê de Ética em Pesquisa em Seres Humanos da Universidade Federal do Ceará
- Comissão Nacional de Ética Em Pesquisa—CONEP
- Comité de ética en la Investigación—CAIMED
- Comité Corporativo de Ética en Investigación Fundación Santafe de Bogotá
- Comité de ética en Investigación Biomédica—CDI
- Comité de Ética en Investigación Biomédica (CEIB) de la Unidad de Investigación Científica de la UNAH.
- Instituto Nacional de Pediatría Comité de Ética en Investigación
- Instituto Nacional de Pediatría
- Comité Ética y de Investigación—UV Universidad Veracruzana
- Saluz Comité de Investigación y Bioética
- Copernicus Group IRB—CGIRB

ClinicalTrials.gov identifiers: NCT01373281; NCT01374516

WHO Universal Trial Numbers: U1111-1116-4957; U1111-1116-4986

Qualified researchers may request access to patient level data and related study documents including the clinical study report, study protocol with any amendments, blank case report form, statistical analysis plan, and dataset specifications. Patient level data will be anonymized and study documents will be redacted to protect the privacy of trial participants. Further details on Sanofi’s data sharing criteria, eligible studies, and process for requesting access can be found at: https://www.clinicalstudydatarequest.com

### Analysis

#### Study cohorts

The CYD14 and CYD15 intent-to-treat (ITT) cohorts consisted of all participants who received at least one injection [9,10]. Analysis of the ITT cohort included all symptomatic, virologically-confirmed dengue events between months 0 and 25 (end of the active follow-up phase). All analyses right-censored participants at month 25 or at dropout if it occurred earlier. The combined CYD14 + CYD15 analyses focused on the age range of the study populations for which CYD-TDV is licensed, 9–16 year-old children. Demographic characteristics of the population combining the two studies are displayed in S1 Table.

#### Descriptive analysis: Attack rates

**Fig 1:**
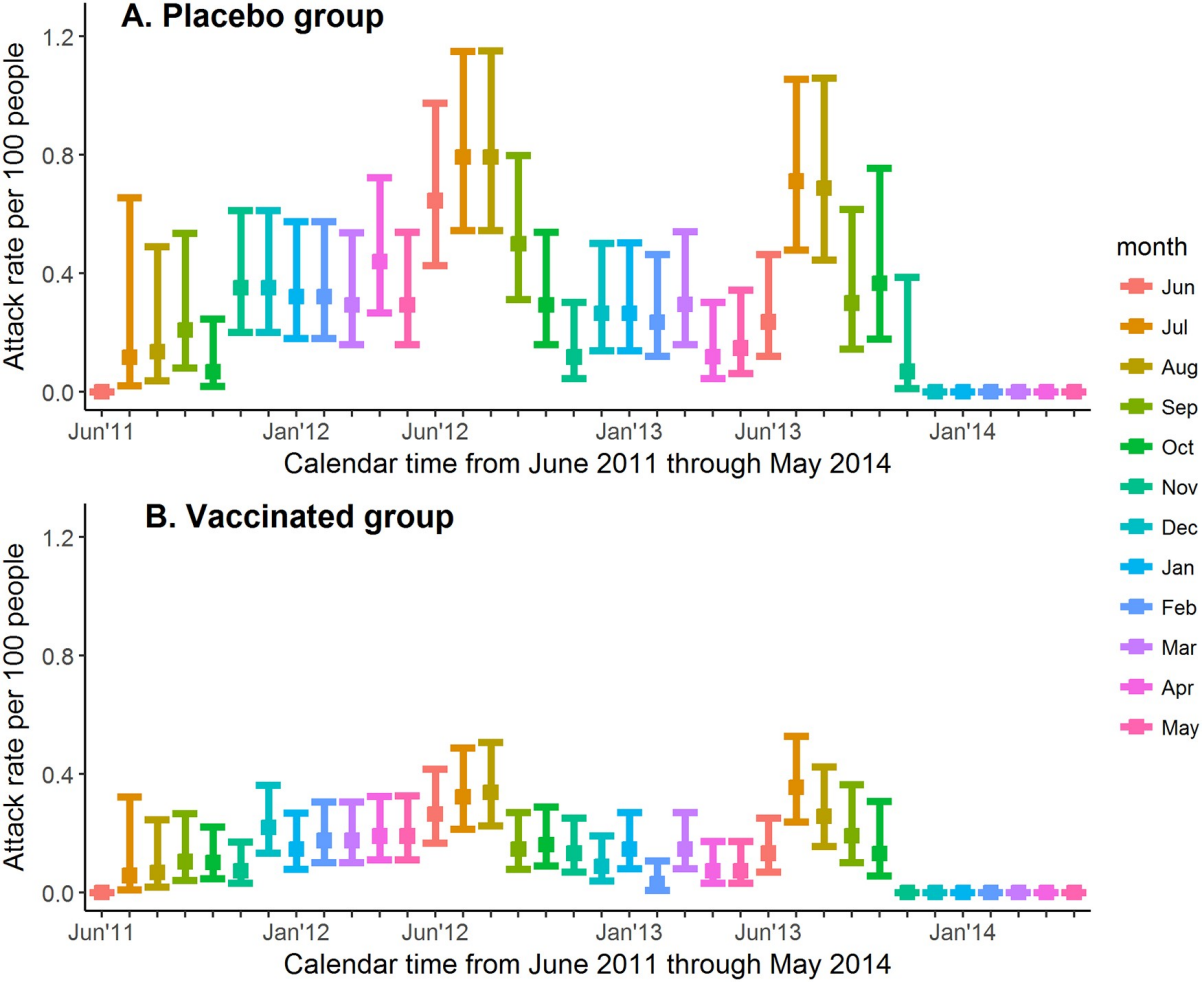
Dengue attack rate by 30-day period in placebo and vaccinated groups of CYD14. (A) Dengue attack rate by 30-day period in placebo group of CYD14. (B) Dengue attack rate by 30-day period in vaccinated group of CYD14.

**Fig 2:**
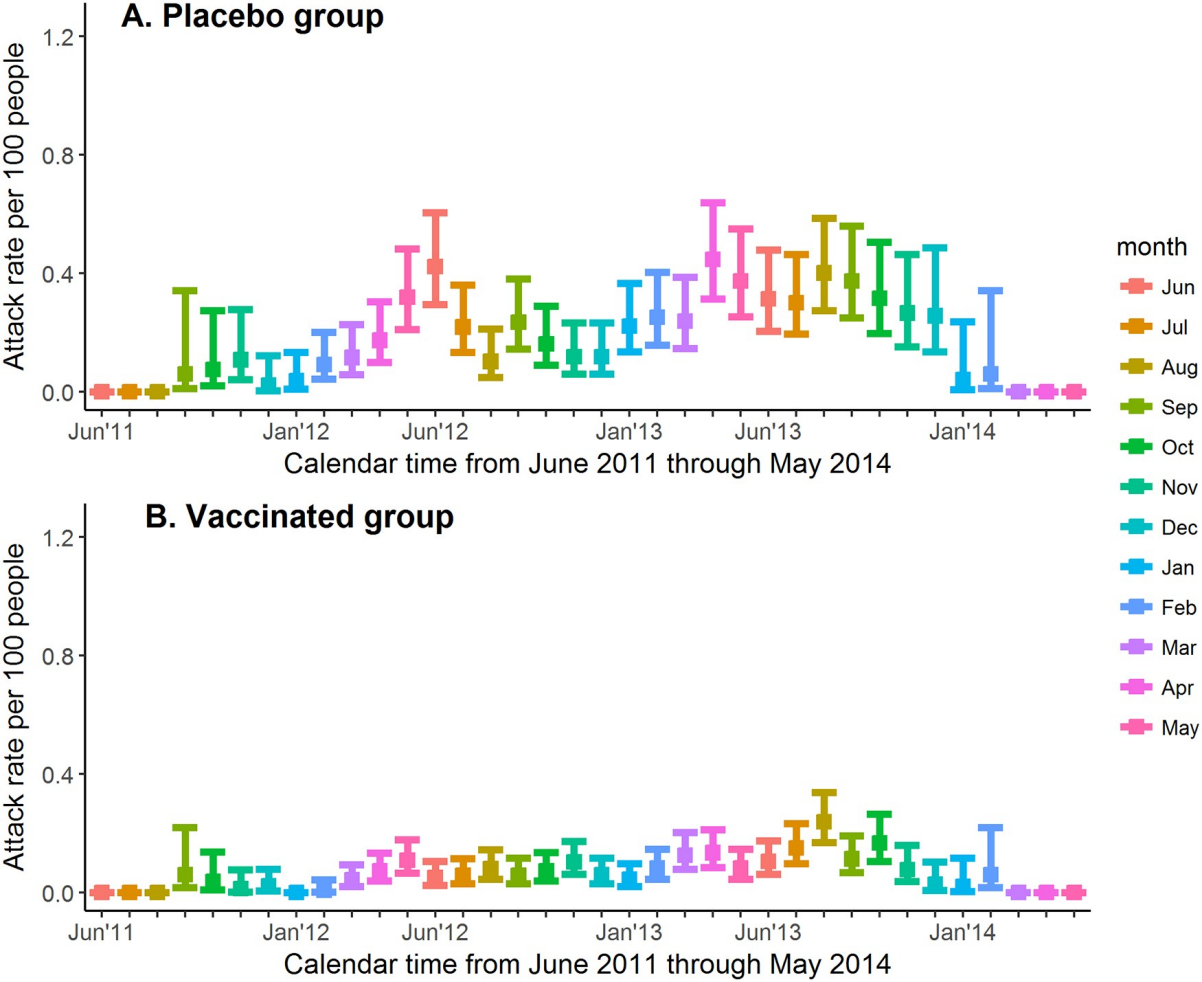
Dengue attack rate by 30-day period in placebo and vaccinated groups of CYD15. (A) Dengue attack rate by 30-day period in placebo group of CYD15. (B) Dengue attack rate by 30-day period in vaccinated group of CYD15.

Plots of the monthly attack rate from June 2011 (the month in which enrollment began) to the end of the active phase follow-up period are shown for the placebo and vaccinated groups of CYD14 (Fig 1) and CYD15 (Fig 2). Months were approximated by 30-day periods. In each month-long interval I, the attack rate was calculated as the number of symptomatic, virologically-confirmed dengue events in I divided by the number of participants at risk in I with 95% confidence interval (CI) calculated by Wilson method of interval estimation for a binomial proportion [42]. Analyses were performed in R Statistical Software [43] version 3.4.3 with the R *binom* package [44]. Participants were at risk for dengue in a given month if the date of enrollment was before the end of that month and the date of their last contact with study staff or the end of the active phase was after the start of that month.

#### Statistical analysis

**Fig 3:**
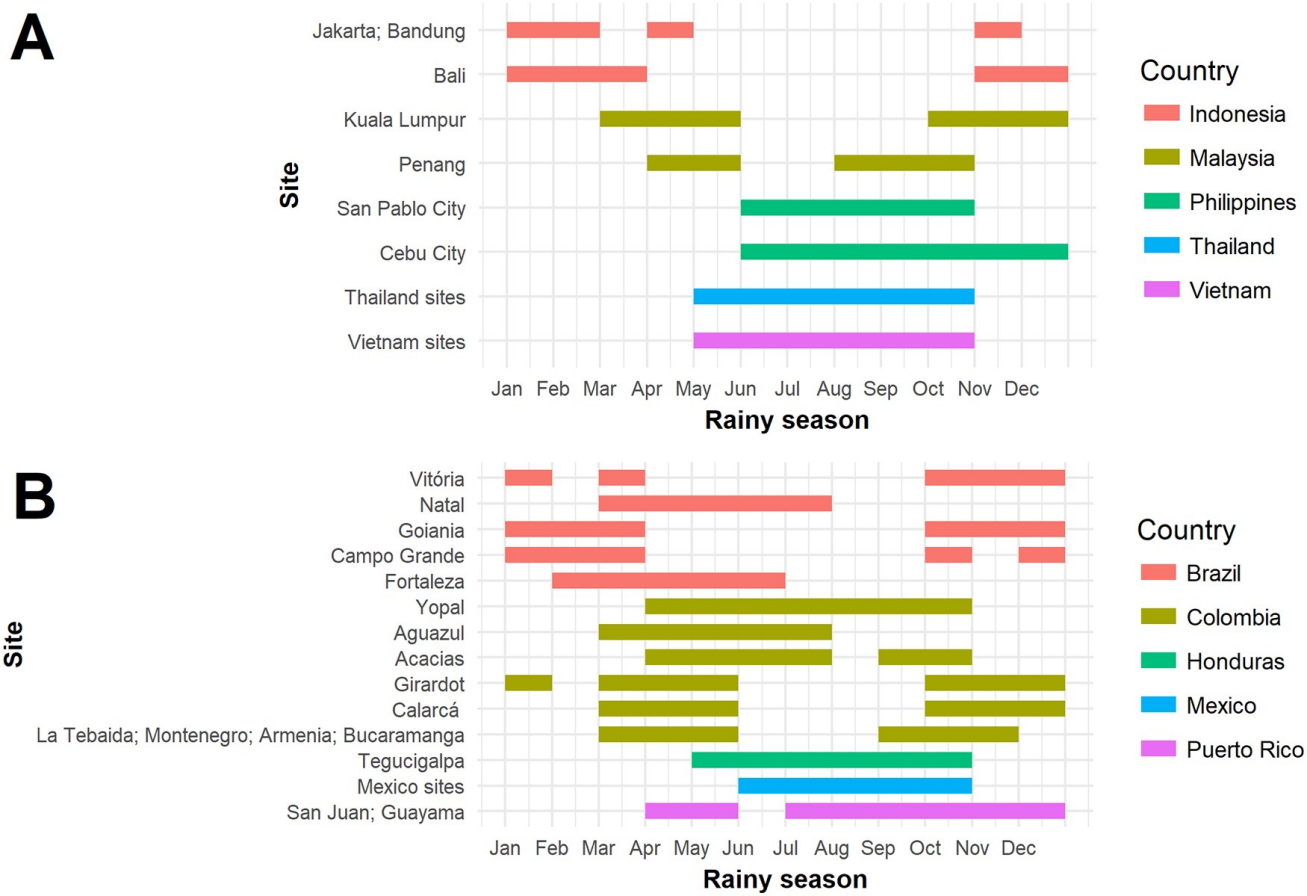
Rainy season of all trials sites (data from World Meteorological Organization). (A) Rainy season of CYD14 sites. (B) Rainy season of CYD15 sites.

Cox proportional hazard models with time-varying covariates were used to determine the association of rainy season with dengue disease and with CYD-TDV efficacy; the proportional hazards assumption was assessed by considering interactions with time and assessing plots of the scaled Schoenfeld residuals. The Cox model was selected to make full use of the time-to-event dengue data and the time-dependent rainy season variable. The rainy season variable was defined as a binary time-dependent variable equal to 1 during the rainy season and 0 otherwise. For each site in each country, the rainy season was determined using climatological information from the World Meteorological Organization (WMO) [45]. This information was based on monthly averages for the 30-year period from 1981 to 2010; contemporaneous rainfall data for the time period in which the trials occurred were not available. If rainy season data were not available on the WMO website, they were obtained from country-specific member websites [46]. Based on this information, the rainy season was defined as all months with average total precipitation (in mm) greater than the average monthly precipitation during the year. The site-specific rainy seasons for CYD14 and CYD15 sites are reported in Fig 3A and 3B, respectively. All models were estimated by adjusting on the baseline covariates sex, protocol-specified age category (2–5, 6–11, 12–14 years in CYD14; 9–11, 12–16 years in CYD15; 9–11, 12–16 years in combined analysis of CYD14 and CYD15), and country (using a nominal categorical variable taking values Indonesia, Malaysia, Philippines, Thailand, and Vietnam in CYD14 and Brazil, Colombia, Honduras, Mexico, and Puerto Rico in CYD15). The expression of the Cox model for the combined analysis of CYD14 and CYD15 is provided in S1 File and a map of the sites is available in S1 Fig. Analyses were performed in R Statistical Software version 3.0.1 with the R *survival* package [47,48]. All p-values are two-sided with significance declared for p-values below 0.05.

## Results

Fig 1 shows the attack rate by month in the placebo (1A) and vaccinated (1B) groups of CYD14 from June 2011 (start of enrollment) to the end of 2013. A seasonal trend can be observed in this plot, with a higher attack rate occurring between June and September in both 2012 and 2013. These months correspond to the rainy seasons of the Philippines (where 49% of the dengue cases occurred), Thailand, and Vietnam. In addition, the proportion of dengue events observed in the vaccine and placebo groups during the rainy season is higher than the proportion of the year that is defined as the rainy season (Table 1). For example, in CYD14 at the Sao Pablo City site in the Philippines, 80% of the total number of dengue cases were observed to occur during the rainy season, which comprised 42% of the year. Fig 2 shows the attack rate by month in the placebo (2A) and vaccinated (2B) groups of CYD15 from June 2011 (start of enrollment) to mid-2014. The seasonal trend observed in CYD15 is less obvious, although more infections appear to have occurred from April-May and September-November. These periods correspond to the rainy seasons of certain sites in Colombia, where 41% of the dengue cases occurred. The seasonal trend in dengue attack rates may be less apparent in CYD15 because the CYD15 sites covered a larger geographical area and the rainy seasons were less similar than in the CYD14 sites (Fig 3A and 3B). The results of the descriptive analysis indicate that our definition of rainy season corresponds to periods of higher observed rates of dengue in CYD14 and in CYD15. Together, these data suggest that rainy season could be a reasonable proxy measurement for periods of increased dengue risk.

**Table 1:** Rainy season (RS) and dengue by country and site in CYD14 and CYD15.

Study	Country	Site	% of the year that is rainy season	No. of dengue events during RS / No. of total dengue events (%)
CYD14	Indonesia	Jakarta	33%	8/29 (28%)
		Bandung	33%	19/39 (49%)
		Bali	42%	9/15 (60%)
	Malaysia	Kuala Lumpur	50%	11/23 (48%)
		Penang	42%	3/7 (43%)
	Philippines	San Pablo City	42%	109/136 (80%)
		Cebu City	58%	113/157 (72%)
	Thailand	Kamphaeng Phet Province;	50%	12/32 (38%)
		Ratchaburi Province	50%	41/57 (72%)
	Vietnam	Long Xuyen	50%	36/59 (61%)
		My Tho	50%	25/41 (61%)
CYD15	Brazil	Vitória	42%	17/30 (57%)
		Natal	42%	27/29 (93%)
		Goiania	50%	14/16 (88%)
		Campo Grande	42%	20/24 (83%)
		Fortaleza	42%	16/20 (83%)
	Colombia	Yopal	58%	52/77 (68%)
		Aguazul	42%	65/78 (83%)
		Acacias	50%	22/43 (51%)
		Girardot	58%	14/22 (64%)
		Calarcá	50%	3/7 (43%)
		La Tebaida	50%	0/0
		Montenegro	50%	0/2
		Armenia	50%	2/5 (40%)
		Bucaramanga	50%	23/38 (61%)
	Honduras	Tegucigalpa	50%	94/113 (83%)
	Mexico	México	42%	60/77 (78%)
		Veracruz	42%	19/22 (86%)
		Tamaulipas	42%	¾ (75%)
		Tizimin	42%	12/16 (75%)
		Valladolid	42%	13/15 (87%)
	Puerto Rico	San Juan	75%	3/8 (38%)
		Guayama	75%	13/16 (82%)

Dengue disease incidence patterns observed in both studies are similar to patterns seen in national dengue surveillance data (Supplemental Material S2.2.4.7, ref [49]). The seasonal trends seen in the monthly attack rates of the placebo groups of each study corresponded with the rainy seasons defined based on the WMO data, suggesting the rainy season represents a period of increased dengue disease. We therefore assumed that if rainfall data had been collected in the studies, the resulting rainy season definitions would not differ substantially from those calculated from the WMO data.

Vaccine efficacy against symptomatic, virologically-confirmed dengue did not change during the rainy season. In CYD14, the estimated VE during the rainy season was 55.8% with 95% CI = (46.9%, 64.6%) and 53.2% with 95% CI = (38.6%, 64.3%) during the non-rainy season. In CYD15, the estimated VE during the rainy season was 64.1% with 95% CI = (57.7%, 70.6%) and 66.7% with 95% CI = (54.8%, 75.5%) during the non-rainy season. The estimated VEs from the models that included rainy season were almost identical to those from previous analyses [9,10]: 54.9% with 95% CI = (47.0%, 61.6%) for CYD14 and 64.8% with 95% CI = (58.9%, 68.8%) for CYD15. The results from the time-dependent Cox models that include or do not include an interaction term for vaccine and rainy season are shown in Tables 2 and 3 for CYD14 and CYD15, respectively. Specifically, the Cox models with time-dependent rainy season showed no significant modification of VE by rainy season in either study and no significant modification in the combined analysis (interaction p-values = 0.74 and 0.69 for CYD14 and CYD15). Rainy season was a strong significant correlate of dengue in the vaccine and placebo groups in each study [hazard ratio (HR) = 2.11 with 95% CI = (1.75, 2.54) in CYD14 and HR = 3.08 with 95% CI = (2.58, 3.67) in CYD15]. Age was also significantly associated with dengue in both CYD14 and CYD15, a finding consistent with previous analyses showing that younger children are at higher risk of symptomatic, virologically-confirmed dengue [9,10]. In addition, country was significantly associated with dengue in both studies (global p-values < 0.01).

**Table 2:** Estimated coefficients, standard errors, hazard ratios and their 95% CIs from time-dependent Cox models in CYD14, with or without the vaccine by rainy season interaction where the interaction term is included to assess effect modification of rainy season on VE.

	Without the Vaccine: Rainy season interaction				With the Vaccine: Rainy season interaction			
	Coefficient estimate (standard error)	Hazard Ratio (95% CI)	P-value	Global p-value^1^	Coefficient (standard error)	Hazard Ratio (95% CI)	P-value	Global p-value^1^
Vaccine	-0.80 (0.082)	0.45 (0.38, 0.53)	< 0.001	-	-0.76 (0.14)	0.47 (0.36, 0.61)^2^	< 0.001	-
Age				< 0.001				< 0.001
2–5	reference	reference	-		reference	reference	-	
6–11	-0.31 (0.092)	0.73 (0.61, 0.88)	< 0.001		-0.31 (0.092)	0.73 (0.61, 0.88)	< 0.001	
12–14	-0.85 (0.13)	0.43 (0.33, 0.55)	< 0.001		-0.85 (0.13)	0.43 (0.33, 0.55)	< 0.001	
Male	-0.064 (0.082)	0.94 (0.80, 1.10)	0.43	-	-0.064 (0.082)	0.94 (0.80, 1.10)	0.43	-
Country				< 0.001				< 0.001
Indonesia	reference	reference	-		reference	reference	-	
Malaysia	-0.79 (0.21)	0.46 (0.30, 0.69)	< 0.001		-0.79 (0.21)	0.46 (0.30, 0.69)	< 0.001	
Philippines	0.57 (0.13)	1.77 (1.38, 2.28)	< 0.001		0.57 (0.13)	1.77 (1.38, 2.28)	< 0.001	
Thailand	0.42 (0.15)	1.52 (1.12, 2.05)	0.007		0.42 (0.15)	1.52 (1.12, 2.05)	0.007	
Vietnam	-0.14 (0.15)	0.87 (0.65, 1.17)	0.36		-0.14 (0.15)	0.87 (0.65, 1.17)	0.36	
Rainy season^-^^3^	0.75 (0.096)	2.11 (1.75, 2.54)	< 0.001	-	0.77 (0.13)	2.17 (1.69, 2.78)	< 0.01	-
Vaccine:Rainy season interaction		-	-	-	-0.056 (0.17)	0.95 (0.67, 1.32)^4^	0.74	-

CI, confidence interval.

^1^For models with categorical variables, the global test assessed whether all the hazard ratios for each category were equal to 1.

^2^Hazard ratio (vaccine/placebo) for the non-rainy season (reference Rainy season category).

^3^Rainy season was site-specific, as defined in Table 1, and the reference category was the non-rainy season.

^4^Hazard ratio (rainy/non-rainy season) for the vaccine group divided by hazard ratio (rainy/non-rainy season) for the placebo group.

**Table 3:** Estimated coefficients, standard errors, hazard ratios and their 95% CIs for time-dependent Cox models in CYD15, with or without the vaccine by rainy season interaction where the interaction term is included to assess effect modification of rainy season on VE.

	Without the Vaccine: Rainy season interaction				With the Vaccine: Rainy season interaction			
	Coefficient estimate (standard error)	Hazard Ratio (95% CI)	P-value	Global p-value^1^	Coefficient estimate (standard error)	Hazard Ratio (95% CI)	P-value	Global p-value^1^
Vaccine	-1.04 (0.079)	0.35 (0.30, 0.41)	< 0.001	-	-1.10 (0.16)	0.33 (0.25, 0.45)^2^	< 0.001	-
Age								
9–11	reference	reference	-		reference	reference	-	
12–16	-0.24 (0.078)	0.79 (0.67, 0.92)	0.002		-0.24 (0.078)	0.79 (0.67, 0.92)	0.002	
Male	0.18 (0.078)	1.20 (1.03, 1.40)	0.02	-	0.18 (0.078)	1.20 (1.03, 1.40)	0.02	-
Country				< 0.001				< 0.001
Brazil	reference	reference	-		reference	reference	-	
Colombia	-0.28 (0.11)	0.75 (0.61, 0.94)	0.01		-0.28 (0.11)	0.76 (0.61, 0.94)	0.01	
Honduras	0.096 (0.13)	1.10 (0.85, 1.43)	0.47		0.096 (0.13)	1.10 (0.85, 1.43)	0.47	
Mexico	0.14 (0.13)	1.15 (0.90, 1.47)	0.28		0.14 (0.13)	1.15 (0.90, 1.47)	0.28	
Puerto Rico	-0.81 (0.22)	0.46 (0.29, 0.69)	< 0.001		-0.81 (0.22)	0.45 (0.29, 0.69)	< 0.001	
Rainy season^3^	1.13 (0.090)	3.08 (2.58, 3.67)	< 0.001	-	1.09 (0.12)	2.99 (2.38, 3.76)	< 0.001	-
Vaccine:Rainy season interaction		-	-	-	0.073 (0.18)	1.08 (0.75, 1.53)^4^	0.69	-

CI, confidence interval.

^1^For models with categorical variables, the global test assessed whether all the hazard ratios for each category were equal to 1.

^2^Hazard ratio (vaccine/placebo) for the non-rainy season (reference Rainy season category).

^3^Rainy season was site-specific, as defined in Table 2, and the reference category was the non-rainy season.

^4^Hazard ratio (rainy/non-rainy season) for the vaccine group divided by hazard ratio (rainy/non-rainy season) for the placebo group.

The results from the combined CYD14 and CYD15 analysis (S2 Table) were consistent with the individual trial analyses: risk of dengue was significantly higher during the rainy season (HR = 2.88 with 95% CI = (2.48, 3.35)) and rainy season did not modify VE (interaction p-value = 0.74). In addition, the effect of rainy season did not differ by study (interaction p-value = 0.60). The combined analysis also showed that study was significantly associated with dengue (p-value < 0.01), with a 30% lower risk of dengue in CYD15 compared to CYD14. This may be explained by the higher risk of dengue associated with the different geographical locations of the studies.

## Discussion

The risk of dengue was significantly higher during the rainy season in the vaccine and placebo groups of each of the phase III trials and in the combined CYD14 and CYD15 analysis, indicating that our definition of rainy season may serve as a proxy for periods of increased dengue transmission. Importantly, CYD-TDV VE did not vary by rainy season in any of the analyses, suggesting that VE is not expected to change during periods of high transmission in endemic regions. One important caveat to be noted is that rainfall data were not collected in the CYD14 and CYD15 studies themselves, but were obtained from publicly available data from the National Meteorological & Hydrological Services in the individual countries. Although these data are likely less accurate than site-level data collection in the trial, the National Meteorological and Hydrological Services data were of sufficient accuracy to define a binary rainy season variable at the site level that we found to be a strong correlate of risk of dengue in both the CYD14 and CYD15 trials, stronger than any other variable considered. However, as the rainy season variable was considered as a binary variable, we are unable to account for the intensity of the rainfall during given time periods. Precipitation can have complex effects on mosquito abundance (and therefore infection force), since moderate rain increases potential breeding grounds but heavy rain may wash out immature mosquito populations [50]. Thus, it may be informative to prospectively collect infection parameters in future studies as they could provide more precise estimates of force of infection than our rainy season variable. In particular, the effect of the volume and/or intensity of the rainy season could be investigated as this would provide information not captured by the dichotomous rainy season variable we considered. Other markers such as placebo incidence or mosquito density could also be considered in the model for measuring the force of infection. Here, rainfall data was used because it could be easily obtained for a public database and we were expected less measurement error in this variable than other measures. Moreover, the existing literature has not established a clear relationship between mosquito density and dengue infection [51]. Still, another potential approach could be to measure mosquito density and consider it as an effect modifier of vaccine efficacy. A more complex analysis could also account for other factors influencing VE. In particular, given the recent findings on the baseline serostatus, the model could be improved by adjusting on this covariate as well.

In the combined analysis of 9–16 years-olds, we found significant different risks of dengue between the studies CYD14 and CYD15; moreover, country was significantly associated with dengue within each study. We investigated whether these findings were due to a difference in the effect of rainy season on dengue in the different age groups of the two studies. We found that no significant effect modification by age on rainy season in either the CYD14 or CYD15 analyses (S3 and S4 Tables respectively), suggesting that the effect of the rainy season was the same on children of all ages within the study ranges. We also performed similar analyses on children aged 9 years and older in CYD14 and found that the increased risk of dengue during the rainy season was similar to that in the all-age CYD14 analysis, suggesting that the increased risk of dengue during the rainy season is similar across the age range in the trials. To more precisely determine how the effect of rainy season varies by geographic location, it would be informative to explore the impact of rainy season within different countries or sites; however, the small number of cases precludes this type of analysis. We hypothesize that observed differences in dengue incidence by site, country, and/or study may reflect different average monthly and/or annual precipitation levels at the different trial sites. A better understanding of the patterns of rainy season on a more local scale would help guide public health policies and would justify strengthened preventive measures at given periods of the year.

Our analyses were performed on the intention-to-treat cohort as this cohort preserves the benefits of randomization and ensures unbiased results. Similar results are expected for the per-protocol cohort for two reasons: first, more than 95% of the subjects in both studies received 3 doses of the vaccine, and then, the VE in the per-protocol cohort has been shown to be nearly identical to VE among participants who had received one or more injections [9,10].

In conclusion, dengue poses a greater risk during the rainy season when large populations of *A*. *aegypti* and *A*. *albopictus* mosquitoes exhibit a high degree of contact with humans. Despite this increase in risk of dengue during the rainy season, there was no evidence to indicate that VE is lower during these periods of increased dengue risk in endemic regions.

## Supporting information

S1 File Cox model equation for the combined analysis. (PDF)

S1 Fig Map of the study sites. Taken from Guy B, Briand O, Lang J, Saville M, Jackson N. Development of the Sanofi Pasteur tetravalent dengue vaccine: One more step forward. Vaccine. 2015 Dec 10;33(50):7100–11.
(TIFF)

S1 Table Demographic characteristics of the population of the CYD14 and CYD15 combined analysis. (PDF)

S2 Table Estimated hazard ratios and 95% CIs for time-dependent Cox models in combined CYD14 and CYD15, without any interaction, with the vaccine by rainall interaction only, with the study by rainfall interaction and with both interactions. (PDF)

S3 Table Estimated hazard ratios and 95% CIs for time-dependent Cox models in CYD14, with or without the age category by rainy season interaction where the interaction term is included to assess effect modification of age on rainy season. (PDF)

S4 Table Estimated hazard ratios and 95% CIs for time-dependent Cox models in CYD15, with or without the age category by rainy season interaction where the interaction term is included to assess effect modification of age on rainy season. (PDF)
