# Supplementary material for: Periods of high dengue transmission defined by rainfall do not impact efficacy of dengue vaccine in regions of endemic disease
Source: PLoS One. 2018 Dec 13;13(12):e0207878. doi: 10.1371/journal.pone.0207878 (PMC6292612; doi:10.1371/journal.pone.0207878)
Supplement: S1 File — (PDF) [file pone.0207878.s001.pdf]

S1 Appendix: Cox model: equation of the hazard function for the combined analysis

$$\lambda(t) = \lambda 0(t)\exp(\beta 1 * Vacc + \beta 2 * Age12 - 14 + \beta 3 * Sex + \beta 4 * Study + \beta 5 * Rainfall + \beta 6 * Vacc * Rainfall + \beta 7 * Study * Rainfall)$$
