# Supplementary figures and images for: Periods of high dengue transmission defined by rainfall do not impact efficacy of dengue vaccine in regions of endemic disease
Source: PLoS One. 2018 Dec 13;13(12):e0207878. doi: 10.1371/journal.pone.0207878 (PMC6292612; doi:10.1371/journal.pone.0207878)

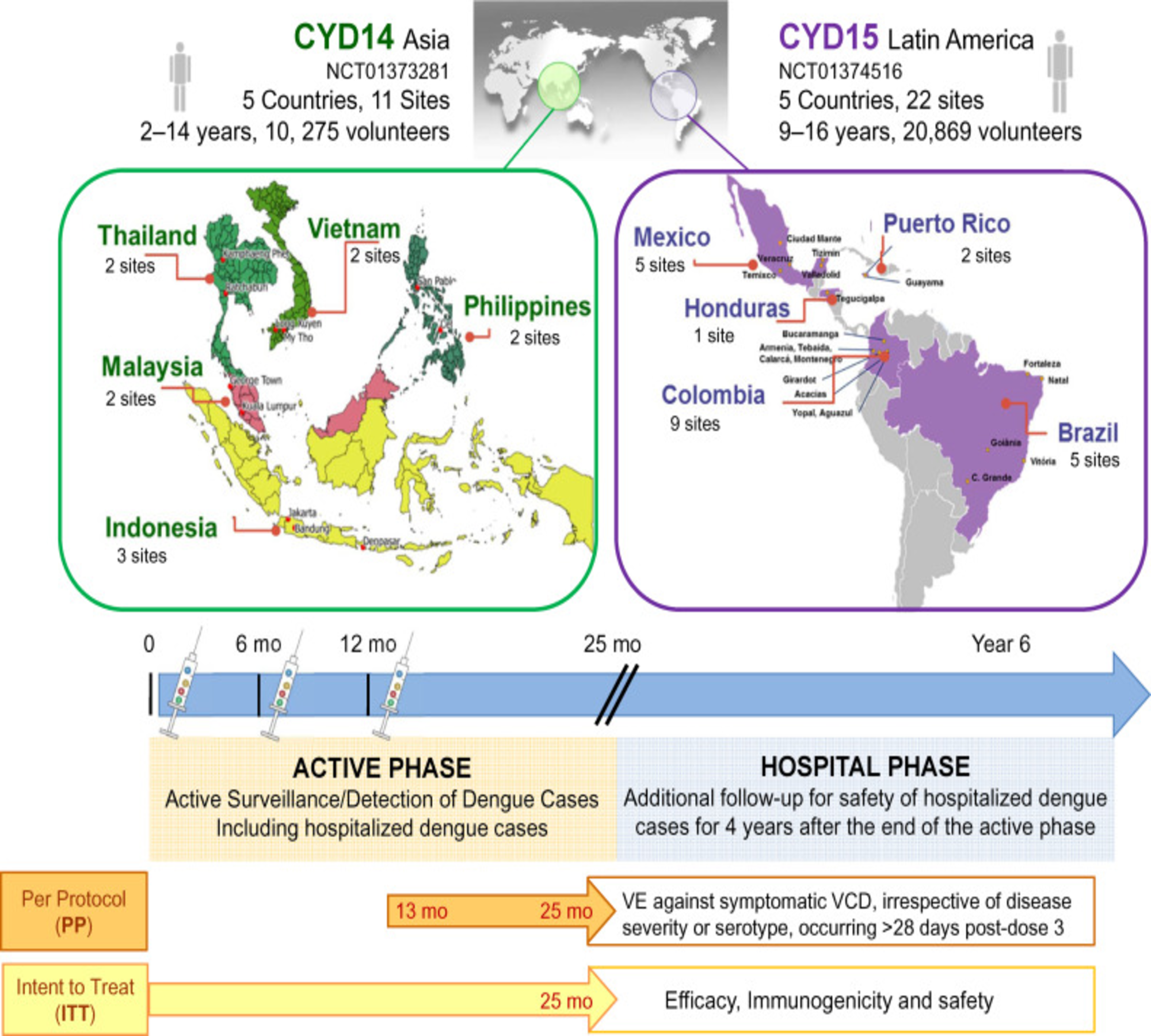

Supplement: S1 Fig — Taken from Guy B, Briand O, Lang J, Saville M, Jackson N. Development of the Sanofi Pasteur tetravalent dengue vaccine: One more step forward. Vaccine. 2015 Dec 10;33(50):7100–11. (TIFF) [file pone.0207878.s002.tiff]
