# Supplementary material for: Periods of high dengue transmission defined by rainfall do not impact efficacy of dengue vaccine in regions of endemic disease
Source: PLoS One. 2018 Dec 13;13(12):e0207878. doi: 10.1371/journal.pone.0207878 (PMC6292612; doi:10.1371/journal.pone.0207878)
Supplement: S1 Table — (PDF) [file pone.0207878.s003.pdf]

S1 Table: Demographic characteristics of the population of the CYD14 + CYD15 combined analysis with children aged 9-16 years

Characteristic	Vaccine group	Control group	Total
Number of participants	17 229 (67%)	8595 (33%)	25 824
Sex			
Male	8480 (49%)	4203 (49%)	12 683 (49%)
Female	8749 (51%)	4392 (51%)	13 141 (51%)
Age group			
9 – 11 yrs	8066 (47%)	4027 (47%)	12 093 (47%)
12 – 16 yrs	9163 (53%)	4568 (53%)	13 731 (53%)
Study			
CYD14	3315 (19%)	1655 (19%)	4970 (19%)
CYD15	13 914 (81%)	6940 (81%)	20 854 (81%)
