# Supplementary material for: Periods of high dengue transmission defined by rainfall do not impact efficacy of dengue vaccine in regions of endemic disease
Source: PLoS One. 2018 Dec 13;13(12):e0207878. doi: 10.1371/journal.pone.0207878 (PMC6292612; doi:10.1371/journal.pone.0207878)
Supplement: S2 Table — (PDF) [file pone.0207878.s004.pdf]

**S2 Table**: Estimated hazard ratios and 95% CIs for time-dependent Cox models in combined CYD14 and CYD15, without any interaction, with the vaccine by rainall interaction only, with the study by rainfall interaction and with both interactions.

| | Without any interaction | | With the vaccine:rainfall interaction only | | With the study:rainfall interaction only | | With both vaccine:rainfall and study:rainfall interactions | |
|---|---|---|---|---|---|---|---|---|
| | Hazard Ratio (95% CI) | P-value | Hazard Ratio (95% CI) | P-value | Hazard Ratio (95% CI) | P-value | Hazard Ratio (95% CI) | P-value |
| Vaccine | 0.34 (0.30, 0.39) | < 0.001 | 0.36 (0.28, 0.46) | < 0.001 | 0.34 (0.30, 0.39) | < 0.001 | 0.36 (0.28, 0.46) | < 0.001 |
| Age<br>9-12<br>12-16 | <br>reference<br>0.74 (0.65, 0.85) | <br>-<br>< 0.001 | <br>reference<br>0.74 (0.65, 0.85) | <br>-<br>< 0.001 | <br>reference<br>0.74 (0.65, 0.85) | <br>-<br>< 0.001 | <br>reference<br>0.74 (0.65, 0.85) | <br>-<br>< 0.001 |
| Male | 1.20 (1.05, 1.37) | 0.008 | 1.20 (1.05, 1.37) | 0.008 | 1.20 (1.05, 1.37) | 0.008 | 1.20 (1.05, 1.37) | 0.008 |
| CYD15 Study | 0.71 (0.61, 0.83) | < 0.001 | 0.71 (0.61, 0.83) | < 0.001 | 0.67 (0.50, 0.88) | < 0.01 | 0.67 (0.50, 0.88) | 0.006 |
| Rainfall | 2.88 (2.48, 3.35) | < 0.001 | 2.94 (2.42, 3.58) | < 0.001 | 2.69 (2.02, 3.60) | < 0.001 | 2.75 (2.01, 3.77) | < 0.001 |
| Vaccine:Rainfall interaction | - | - | 0.95 (0.70, 1.28) | 0.74 | - | - | 0.95 (0.70, 1.28) | 0.74 |
| Study:Rainfall interaction | - | - | - | - | 1.09 (0.78, 1.53) | 0.60 | 1.09 (0.78, 1.53) | 0.60 |
