# Supplementary material for: Periods of high dengue transmission defined by rainfall do not impact efficacy of dengue vaccine in regions of endemic disease
Source: PLoS One. 2018 Dec 13;13(12):e0207878. doi: 10.1371/journal.pone.0207878 (PMC6292612; doi:10.1371/journal.pone.0207878)
Supplement: S3 Table — (PDF) [file pone.0207878.s005.pdf]

S3 Table: Estimated hazard ratios and 95% CIs for time-dependent Cox models in CYD14, with or without the age category by rainy season interaction where the interaction term is included to assess effect modification of age on rainy season.

	Without the Age category:Rainy season interaction			With the Age category:Rainy season interaction		
	Hazard Ratio (95% CI)	P-value	Global p-value	Hazard Ratio (95% CI)	P-value	Global p-value
Vaccine	0.45 (0.38, 0.53)	< 0.001	-	0.45 (0.38, 0.53)	< 0.001	-
Age			< 0.001			< 0.001
2-5	reference	-		reference	-	
6-11	0.73 (0.61, 0.88)	< 0.001		0.76 (0.56, 1.02)	0.06	
12-14	0.43 (0.33, 0.55)	< 0.001		0.43 (0.33, 0.55)	< 0.001	
Male	0.94 (0.80, 1.10)	0.43	-	0.94 (0.80, 1.10)	0.43	-
Country			< 0.001			< 0.001
Indonesia	reference	-		reference	-	
Malaysia	0.46 (0.30, 0.69)	< 0.001		0.46 (0.30, 0.69)	< 0.001	
Philippines	1.77 (1.38, 2.28)	< 0.001		1.77 (1.38, 2.28)	< 0.001	
Thailand	1.52 (1.12, 2.05)	0.007		1.52 (1.12, 2.05)	0.007	
Vietnam	0.87 (0.65, 1.17)	0.36		0.87 (0.65, 1.17)	0.38	
Rainy season	2.11 (1.75, 2.54)	< 0.001	-	2.05 (1.53, 2.75)	< 0.001	-
6-11yrs:Rainy season interaction	-	-	-	0.95 (0.66, 1.37)	0.79	0.21
12-14yrs: Rainy season interaction	-	-	-	1.54 (0.88, 2.71)		-
